# F1534S *kdr* mutation drives the geographical heterogeneity of pyrethroid resistance in *Aedes albopictus* from Hangzhou, China

**DOI:** 10.3389/fcimb.2026.1777964

**Published:** 2026-03-16

**Authors:** Binbin Jin, Jiawen Du, Lingya Wei, Tianxiao Duan, Jiabao Xu, Yinghong Wang, Ye Lu, Hui Jin

**Affiliations:** 1 Institute of Disinfection and Vector Control, Hangzhou Center for Disease Control and Prevention (Hangzhou Health Supervision Institution), Hangzhou, Zhejiang, China; 2 Zhejiang Key Laboratory of Multi-Omics in Infection and Immunity, Westlake University, Hangzhou, China; 3 School of Laboratory Medicine and Life Sciences, Wenzhou Medical University, Wenzhou, Zhejiang, China; 4 Department of Quality Management, Hangzhou Center for Disease Control and Prevention (Hangzhou Health Supervision Institution), Hangzhou, Zhejiang, China; 5 School of Basic Medical Sciences, Zhejiang Chinese Medical University, Hangzhou, Zhejiang, China; 6 Environmental and Health Institute, Zhejiang Provincial Center for Disease Control and Prevention, Hangzhou, Zhejiang, China

**Keywords:** *Aedes albopictus*, F1534S, Hangzhou, insecticide resistance, knockdown resistance (*kdr*), voltage-gated sodium channel (VGSC)

## Abstract

**Introduction:**

The rapid evolution of insecticide resistance in mosquitoes has substantially compromised control efficacy against *Aedes albopictus*. This study aimed to evaluate the resistance levels to commonly used insecticides in field populations of *Ae. albopictus* from Hangzhou, China, and characterize knockdown resistance (*kdr*) mutations in the *voltage-gated sodium channel* (*VGSC*) gene, thereby providing a scientific basis for developing localized insecticide resistance management strategies.

**Methods:**

Field populations of *Ae. albopictus* were collected from 14 districts in Hangzhou from May to September 2025. Resistance to 0.2% fenitrothion, 0.2% bendiocarb, 1.4% alpha-cypermethrin, 0.4% permethrin, and 0.7% beta-cyfluthrin was evaluated using the adult mosquito contact tube bioassays. Mutations in multiple *kdr* loci within VGSC domains and the G119 site of the *ace-1* gene were detected by PCR amplification followed by Sanger sequencing.

**Results:**

Bioassays revealed that all field populations were resistant to permethrin (mortality: 2.67%–88.00%), and 86.7% of populations exhibited resistance or possible resistance to beta-cyfluthrin. However, all populations were susceptible to bendiocarb, and no confirmed resistance to fenitrothion was detected. Molecular analyses identified a high-frequency mutation exclusively at the F1534 locus of the *VGSC* gene (mutant TCC allele frequency: 89.23%). Significant variations in genotypic frequency distribution were observed among the districts (p < 0.001). Core urban districts (e.g., Gongshu and Yuhang) exhibited significantly higher *kdr* allele frequencies (>98%) and resistance levels compared with counties such as Fuyang and Lin’an.

**Conclusion:**

*Ae. albopictus* populations in Hangzhou exhibit widespread resistance to pyrethroid insecticides, which is spatially associated with a high frequency of the F1534S *kdr* mutation in the *VGSC* gene. The potential contribution of metabolic resistance, which may vary geographically, was not evaluated in this study. These findings suggest that resistance management strategies, including insecticide rotation and area-specific interventions, may be needed and should be informed by further investigation of local resistance mechanisms.

## 1 Introduction

The Asian tiger mosquito (*Aedes albopictus*) is an invasive vector of major public health relevance, serving as a primary transmitter for dengue, chikungunya, and Zika viruses. Its exceptional ecological plasticity and rapid global expansion have made it a key target for vector control in southern China, including the subtropical regions (Benedict et al., 2007; Li et al., 2014). Hangzhou, a major city in the Yangtze River Delta, provides favorable climatic and environmental conditions for the proliferation of *Ae. albopictus*. Moreover, rapid urbanization and climate change have led to increased mosquito densities (Ryan et al., 2019; Yue et al., 2018), consequently elevating the risk of mosquito-borne disease transmission and presenting a significant challenge to regional public health.

Chemical control remains the primary means of *Ae. albopictus* management. Pyrethroid insecticides are predominantly employed owing to their high efficacy and relatively low mammalian toxicity (Ahamad and Kumar, 2023). However, their intensive and long-term use has led to the evolution of widespread resistance in *Ae. albopictus* populations across China (Yang et al., 2021). Several studies from southern and eastern China report elevated resistance ratios (RR_50_ >10) and low mortality rates (typically well below 90% at diagnostic doses) to pyrethroids, indicating that resistance has become a key obstacle to effective vector control (Li et al., 2021; Zheng et al., 2022). Furthermore, resistance levels vary significantly across different geographical populations, likely reflecting localized insecticide selection pressures and usage practices (Su et al., 2019; Zheng et al., 2022).

At the molecular level, insecticide resistance in *Ae. albopictus* is primarily driven by target-site mutations and enhanced metabolic detoxification. Notably, knockdown resistance (*kdr*) mutations in the *voltage-gated sodium channel* (*VGSC*) gene are the most extensively characterized mechanism underlying pyrethroid resistance (Rinkevich et al., 2013). Various *kdr* mutations, including F1534S, F1534C, and V1016G, have been observed in *Ae. albopictus* populations across China and globally (Moyes et al., 2017; Yuan et al., 2023; Zhao et al., 2023; Zheng et al., 2022). These mutations reduce the binding affinity of pyrethroid to *VGSC*, thereby diminishing insecticide efficacy. Simultaneously, the upregulation of metabolic enzymes, including cytochrome P450 monooxygenases, carboxylesterases, and glutathione S-transferases, also plays a role in resistance through enhanced insecticide degradation (Liu, 2015). The relative importance of these metabolic mechanisms appears to vary substantially among populations, and their predominant role remains unresolved (Moyes et al., 2017). Therefore, this study primarily focuses on *kdr* mutations to elucidate the genetic basis of pyrethroid resistance in local *Ae. albopictus* populations.

Despite frequent insecticide use and the complex population dynamics of *Ae. albopictus* in Hangzhou, systematic data on resistance levels and associated *kdr* mutations remain limited and lack comprehensive geographical coverage. Although our previous preliminary monitoring in selected urban areas revealed the presence of *kdr* mutations (Jin et al., 2025), it was insufficient to encompass all districts and guide city-wide targeted insecticide use strategies. Therefore, a comprehensive, city-wide assessment of insecticide resistance and its underlying mechanisms is urgently needed. To address this, this study aims to integrate bioassay-based resistance monitoring with molecular genetic analyses to comprehensively characterize the current insecticide resistance status and *kdr* genotype diversity of *Ae. albopictus* populations in Hangzhou. These findings will provide a critical scientific basis for optimizing insecticide selection and formulating localized, evidence-based resistance management strategies, ultimately improving the precision and effectiveness of vector control and public health protection in the region.

## 2 Materials and methods

### 2.1 Study site selection and characteristics

To analyze the spatial heterogeneity of resistance patterns across the city, a total of 14 administrative districts, including, urban core, suburban, county, and county-level city areas were selected for mosquito sampling. Site selection followed a stratified approach designed to capture key gradient parameters that may influence insecticide selection pressure. This included urbanization intensity, ranging from densely populated urban cores (e.g., Shangcheng and Gongshu Districts) to rapidly developing peri−urban zones (e.g., Yuhang and Qiantang Districts), and outer counties with more agricultural or natural landscapes (e.g., Fuyang, Lin’an, Tonglu, and Chun’an). The other parameters were the presumed insecticide application pressure, based on historical records and routine practices of local vector control departments, with core urban areas typically expected to implement more frequent and widespread pyrethroid−based space spraying in response to epidemics, along with breeding habitat composition variation, with artificial containers (e.g., household water−holders, discarded tires) being predominant in urban sites, while a larger number of natural water bodies being prevalent in peri−urban and rural sites.

### 2.2 Mosquito collection and rearing

Field populations of *Ae. albopictus* were collected from the 14 districts across Hangzhou, China, from May to September 2025. Both adults and larvae were sampled (**Supplementary Table 1**, **Supplementary Figure 1**). Adult mosquitoes were collected using BG-Sentinel traps (Biogents AG, Regensburg, Germany) and double-layer mosquito nets. Following morphological identification, female *Ae. albopictus* adults were stored separately according to the collection site at -80°C for subsequent DNA extraction.

Larvae were collected from natural and artificial water containers, including outdoor water-holding vessels, discarded tires, and flowerpot saucers, using a pipette method. The collected larvae were transported to the insectary of the Hangzhou Center for Disease Control and Prevention, maintained in plastic pans, and fed turtle food powder (INCH-GOLD, Shenzhen, China). Rearing conditions were maintained at a constant temperature of 27 ± 2°C, relative humidity of 70% ± 10%, and a photoperiod of 14-h light: 10-h dark. Upon emergence, adults were housed in 30×30×30 cm nylon mesh cages and provided free access to a 10% glucose solution. Female mosquitoes were blood-fed with defibrinated sheep blood (Solarbio Life Sciences, Beijing, China) delivered via a Hemotek membrane feeding system (Discovery Workshops, Accrington, UK).

A susceptible reference strain of *Aedes albopictus* (designated as Lab) was used as a control in all bioassays and molecular analyses. This strain, developed at the Chinese Center for Disease Control and Prevention (China CDC) was provided to our laboratory by the Zhejiang CDC. It has been maintained in the insectary of the Hangzhou CDC for over 200 generations under the standard rearing conditions described above, with no exposure to any insecticides in order to preserve its susceptibility.

### 2.3 Insecticide susceptibility bioassay

Insecticide susceptibility in F1 generation female adults (3–5 days old) was assessed using the adult mosquito contact tube bioassays according to the Chinese National Standard GB/T 26347-2010 (Mosquito insecticide resistance bioassay methods). According to this standard, mortality ≥ 98% is categorized as Susceptible (S), mortality between 80% and 97% is categorized as Possibly Resistant (PR), and mortality < 80% is categorized as Resistant (R). This national standard was chosen to ensure consistency with domestic vector surveillance programs and facilitate comparison with previous studies conducted in China. The diagnostic concentrations tested were 0.2% fenitrothion (organophosphate), 0.2% bendiocarb (carbamate), 1.4% alpha-cypermethrin, 0.4% permethrin, and 0.7% beta-cyfluthrin (pyrethroids). All insecticide-impregnated papers were supplied by the National Institute for Communicable Disease Control and Prevention, China CDC.

For each field population and each insecticide, 60–75 F1 female adults (3–5 days old, non-blood-fed) that were divided into three independent replicates of 20–25 mosquitoes per replicate, were tested. In every replicate test, mosquitoes were introduced into adult mosquito contact tube lined with insecticide−impregnated papers at the diagnostic concentrations described above. They were subsequently transferred to clean holding tubes containing cotton wool soaked in 10% glucose solution post 1 h of exposure, and mortality was recorded at 24 h post−exposure. A control group exposed to solvent−only treated papers was included in every replicate test. Since the control mortality was consistently <5%, Abbott’s correction was not applied. Additionally, although not observed in this study, any test with a control mortality exceeding 20% would need to be discarded and repeated.

### 2.4 DNA extraction and *kdr* genotyping

Genomic DNA was extracted from individual adult mosquitoes collected from the field at each of the 14 districts using the QIAamp DNA Blood & Tissue Kit (QIAGEN, Germany), according to the manufacturer’s instructions. The number of mosquitoes successfully genotyped per district varied from 3 to 39, with a total of 289 individuals (**Table 1**). DNA concentration and purity were measured using a NanoPhotometer NP80 (IMPLEN, USA), and qualified samples were stored at -20°C for subsequent analyses.

**Table 2:** Amplification products and reaction conditions for *Ae. albopictus VGSC* gene and *AChE* gene fragments.

Gene	Amplify fragments	Primer name	Mutation sites	Primer sequences (5’ - 3’)	Annealing temperature (°C)	Product length (bp)
*VGSC*	Domains II	acgSCF20	S989、I1011、L1014、V1016	GACAATGTGGATCGCTTCCC	55	480
		acgSCR21		GCAATCTGGCTTGTTAACTTG		
	Domains III	acgSCF7	I1532、F1534	GAGAACTCGCCGATGAACTT	53	740
		acgSCR7		GACGACGAAATCGAACAGGT		
	Domains IV	acgSCF6	D1763	TCGAGAAGTACTTCGTGTCG	55	280
		acgSCR8		AACAGCAGGATCATGCTCTG		
*AChE*	*Ace-1*	L119	G119S	CTGTTCGAATTGTAGATGCCGA	55	701
		R119				

Key *kdr* loci within the *VGSC* gene, namely S989, I1011, L1014 (Domain II), I1532, F1534 (Domain III), and D1763 (Domain IV), as well as the G119 site in the acetylcholinesterase gene (*ace-1*), were amplified by PCR using previously validated primers (**Table 2**) (Caiying et al., 2020; Kasai et al., 2011). PCR amplification was performed in a 50 μL reaction volume containing 25 μL of 2× PCR Master Mix, 2 μL of each forward and reverse primer (10 μM), 2 μL of template DNA, and nuclease-free water up to a final volume of 50 μL. The primer sequences and thermal cycling conditions are listed in **Table 1**. The PCR products were visualized on 1.5% agarose gels and sent to Sangon Biotech (Shanghai, China) for bidirectional Sanger sequencing. The resulting sequences were aligned and analyzed using MEGA 11.0.13 software (Tamura et al., 2021), and the chromatograms were inspected using Chromas 2.6.6 software (Technelysium Pty. Ltd., Queensland, Australia) to determine genotypes at each locus.

**Table 1:** *Kdr* mutations at the F1534 site of the *VGSC* gene in *Ae. Albopictus* from different districts of Hangzhou.

Administrative division	Abbreviation of administrative region	Number of adult mosquitoes (pieces)	Genotype [pieces (%)]			Allele frequency (%)	
			F1534F (TTC)	F1534F/S (TTC/TCC)	F1534S (TCC)	TTC	TCC
Shangcheng District	SC	18	0 (0.00)	2 (11.11)	16 (88.89)	5.555	94.445
Gongshu District	GS	28	0 (0.00)	1 (3.57)	27 (96.43)	1.785	98.215
Xihu District	XH	19	0 (0.00)	1 (5.26)	18 (94.74)	2.630	97.370
Binjiang District	BJ	27	2 (7.41)	3 (11.11)	22 (81.48)	12.965	87.035
Xiaoshan District	XS	26	0 (0.00)	5 (19.23)	21 (80.77)	9.615	90.385
Yuhang District	YH	26	0 (0.00)	1 (3.85)	25 (96.15)	1.925	98.075
Fuyang District	FY^*^	8	1 (12.50)	4 (50.00)	3 (37.50)	37.500	62.500
Lin’an District	LA	20	3 (15.00)	4 (20.00)	13 (65.00)	25.000	75.000
Linping District	LP	23	2 (8.70)	4 (17.39)	17 (73.91)	17.395	82.605
Qiantang District	QT	28	0 (0.00)	2 (7.14)	26 (92.86)	3.570	96.430
Tonglu County	TL^*^	3	0 (0.00)	0 (0.00)	3 (100.00)	0.000	100.000
Chun’an County	CA^*^	6	0 (0.00)	1 (16.67)	5 (83.33)	8.335	91.665
Jiande City	JD	39	7 (17.95)	5 (12.82)	27 (69.23)	24.360	75.640
West Lake Scenic Area	WLSA	26	0 (0.00)	1 (3.85)	25 (96.15)	1.925	98.075
Control	Lab	8	8 (100.00)	0 (0.00)	0 (0.00)	100.000	0.000
Total (exclude the control)		297	15 (5.05)	34 (11.45)	248 (83.50)	10.775	89.225

F/S represent the amino acids encoded by the two alleles at position 1534 (F1534) of the *VGSC* gene, F denotes Phenylalanine, S denotes Serine. Allele frequency = (2 × homozygous mutants + heterozygotes)/ (2 × total individuals). F1534F represents the wild-type homozygous genotype, F1534F/S (TTC/TCC) represents the mutant heterozygous genotype, and F1534S (TCC) represents the mutant homozygous genotype.

*In some administrative regions, due to the difficulty in capturing samples and the small sample size (such as TL County n=3), the allele frequency estimates should be interpreted with caution.

*kdr* genotyping was performed in adult females collected from the field at each sampling site. The mosquitoes used in the insecticide bioassays were the F1 progeny of larvae collected from various habitats within these same sampling sites. While not comprising the exact same individuals, both sets of samples originated from the same local *Ae. albopictus* populations, thus ensuring that the genotypic data are representative of the observed phenotypic resistance.

### 2.5 Statistical analysis

All statistical analyses were performed using SPSS 20.0 software. Mortality data from bioassays were expressed as percentages. Differences in mortality rates among different district populations were compared using the Kruskal-Wallis H test, followed by Dunn’s *post hoc* test with Bonferroni correction, as the data did not meet the assumptions of normality and homogeneity of variance. The association between the *kdr* mutation and phenotypic resistance at the population level was assessed by conducting a Pearson’s correlation analysis between the district-level mutant allele (TCC) frequency at the F1534 locus, and the corresponding mortality rates for each pyrethroid insecticide (1.4% alpha-cypermethrin, 0.4% permethrin, and 0.7% beta-cyfluthrin).The distribution of resistance levels (susceptible/possibly resistant/resistant) and genotype frequencies among populations was compared using the chi-square (χ²) test or Fisher’s exact test when the expected frequencies were less than five. A p-value < 0.05 was considered statistically significant for all analyses.

Mortality percentages were reported as observed values without Abbott’s correction since control mortality in all assays were below 5%.

## 3 Results

### 3.1 Phenotypic insecticide resistance in *Ae. albopictus*

**Figure 1:**
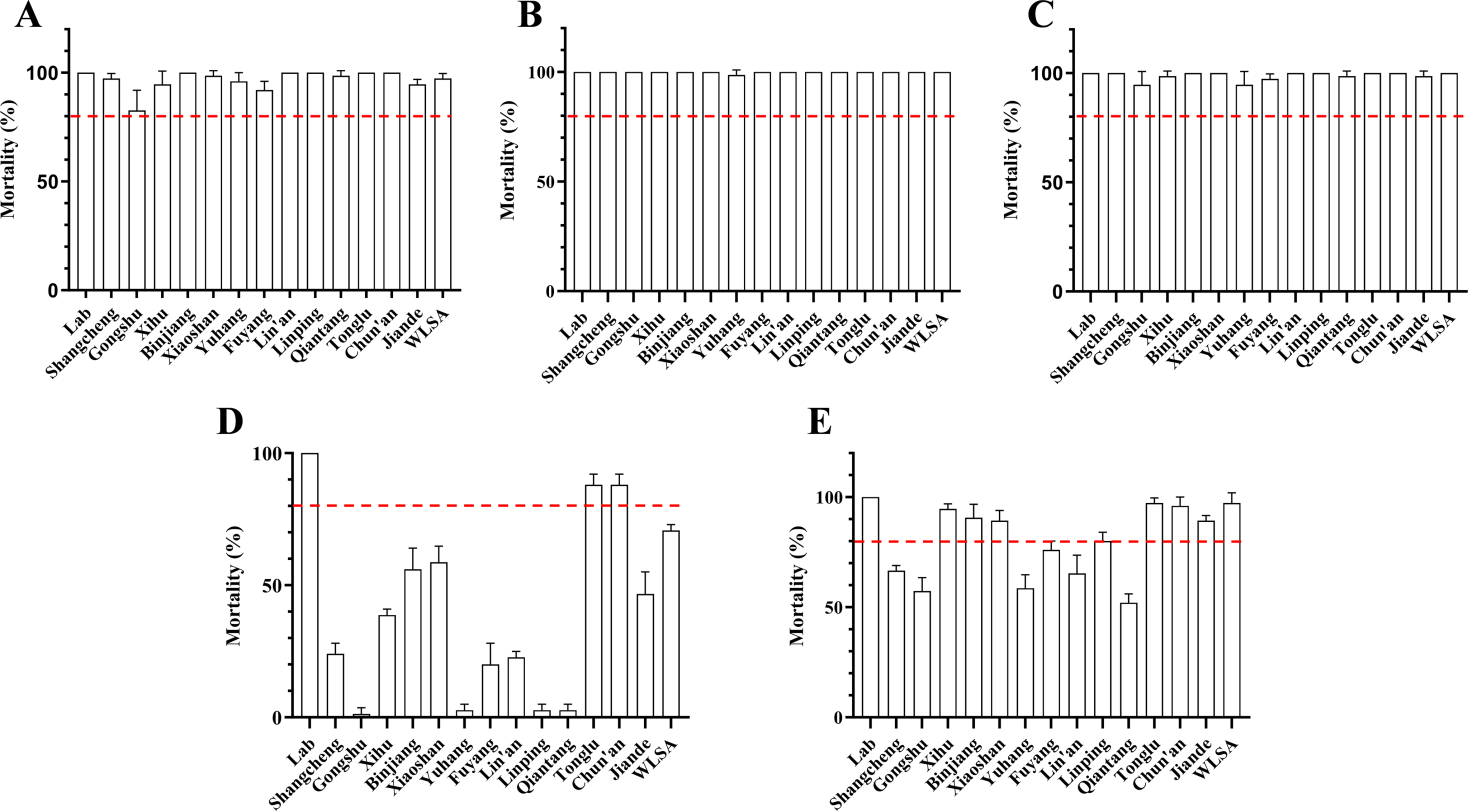
Resistance levels of *Ae. albopictus* populations to five insecticides across various districts of Hangzhou. This figure illustrates the insecticide resistance status of field-collected *Ae. albopictus* populations from 14 administrative districts in Hangzhou, assessed using the adult mosquito contact tube bioassays. Results are expressed as the mortality (%), and resistance status was classified relative to a laboratory-susceptible reference strain (Lab). The panels show the mortality rates of various insecticides: **(A)** 0.2% fenitrothion (organophosphate). **(B)** 0.2% bendiocarb (carbamate). **(C)** 1.4% alpha-cypermethrin (pyrethroid). **(D)** 0.4% permethrin (pyrethroid). **(E)** 0.7% beta-cyfluthrin (pyrethroid). Abbreviations on the x-axis of the graphs correspond to field populations from various districts of Hangzhou City; the full district names are provided in the “Administrative division” column of the **Table 1**. “Lab” denotes the laboratory-susceptible strain used as the susceptible baseline control. Each bar represents the mean mortality rate, and the error bars represent the standard deviation (SD) of three replicate experiments. The red dashed line represents the 80% mortality threshold, used to distinguish between ‘potentially resistant’ (80–97%) and ‘resistant’ (<80%) categories. Resistance status was classified according to the Chinese National Standard GB/T 26347-2010: mortality ≥ 98% is Susceptible (S), 80–97% is Possibly Resistant (PR), and <80% is Resistant (R). Detailed mortality values and resistance classifications for each population are provided in **Supplementary Table 2**.

All evaluated field populations (14/14) exhibited mortality rates below 97%, with the lowest and highest observed mortalities being 1.33% (Gongshu District) and 88.00% (Tonglu County), respectively, on exposure to 0.4% permethrin, thus confirming resistance. Except for Tonglu County (TL) and Chun’an County (CA), which were classified as “possibly resistant,” the remaining 12 populations were categorized as “resistant.” For beta-cyfluthrin, mortality ranged from 52.00% to 97.33%, with 6 and 8 out of the 14 populations classified as “resistant” and “possibly resistant”, respectively. The highest susceptibility was observed to alpha-cypermethrin, with mortality ranging from 94.67% to 100%, with 78.57% (11/14) of populations classified as “susceptible” to the insecticide (**Figure 1**, **Supplementary Table 2**). Chi-square tests revealed significant differences in resistance distribution among districts for all three pyrethroids (permethrin: χ² = 80.4, df = 13, p < 0.001; beta-cyfluthrin: χ² = 52.6, df = 13, p < 0.001; and alpha-cypermethrin: χ² = 45.2, df = 13, p < 0.001).

Exposure to fenitrothion produced mortality rates ranging from 82.67% to 100%. None of the tested populations met the “resistant” threshold; 50% (7/14) were classified as “susceptible” and 50% (7/14) as “possibly resistant.” Furthermore, significant geographical variation in malathion susceptibility was also observed (χ² = 35.8; df = 13, p = 0.003). All tested populations (15/15) were fully susceptible to 0.2% chlorfenapyr, with mortality ranging from 98.67% to 100%.

Kruskal-Wallis H tests revealed significant differences in mortality among districts for permethrin (H = 45.2; df = 13, p < 0.001) and beta-cyfluthrin (H = 38.1; p = 0.001). *Post-hoc* pairwise comparisons (Dunn’s test with Bonferroni correction) revealed that core urban districts, namely Gongshu (GS), Yuhang (YH), and Qiantang (QT), exhibited significantly lower mortality to both insecticides compared with peripheral counties such as TL and CA (adjusted p < 0.05). The laboratory-susceptible strain (Lab) exhibited 100% mortality to all tested insecticides and was uniformly classified as “susceptible.”

To elucidate the underlying molecular mechanisms, we further characterized mutations in the target-site genes *vgsc* and *ace-1*.

### 3.2 Allele frequency of *Ace1* and *Kdr* mutations

To elucidate the molecular mechanisms underlying target-site resistance to pyrethroid insecticides in *Ae. albopictus* populations from Hangzhou, we screened key mutation sites in the *vgsc* and *ace-1* genes. No mutant alleles were detected at the following loci: S989, I1011, and L1014 in domain II; I1532 in domain III; D1763 in domain IV of *VGSC*; or at G119 in *ace-1*.

A nonsynonymous mutation from TTC (phenylalanine, F) to TCC (serine, S) was identified at codon F1534 in domain III of the *VGSC* gene. Genotyping results for this locus (**Table 1**) revealed that the mutant homozygous genotype (F1534S) was predominant among 297 field-collected individuals (excluding the laboratory-susceptible strain), with a frequency of 83.50% (248/297). The frequencies of the wild-type homozygous (F1534F) and heterozygous (F1534F/S) genotypes were 5.05% (15/297) and 11.45% (34/297), respectively. Consequently, the overall mutant allele (TCC) frequency was 89.23%.

Chi-square goodness-of-fit tests indicated that the observed genotype frequencies in the pooled field populations significantly deviated from Hardy-Weinberg equilibrium expectations (χ² = 415.3, df = 1, p < 0.001), suggesting strong selective pressure. Per−population HWE testing was not performed due to the insufficient sample sizes in several districts. Fisher’s exact tests (applied owing to expected counts < 5 in some categories) revealed highly significant differences in genotype distribution among geographic populations (p < 0.001). *Post-hoc* pairwise comparisons with Bonferroni correction demonstrated that genotype profiles of populations from Fuyang (FY), Lin’an (LA), and Jiande (JD) differed markedly compared with those from Shangcheng (SC), Gongshu (GS), Yuhang (YH), and the West Lake Scenic Area (WLSA) (adjusted p < 0.05). Notably, the frequency of mutant homozygotes in FY (37.50%; 3/8) was significantly lower than that in GS (96.43%; 27/28) and YH (96.15%; 25/26). Correspondingly, mutant allele frequencies in FY (62.50%), LA (75.00%), and JD (75.64%) were significantly lower than those in GS (98.21%), YH (98.08%), and WLSA (98.08%) (adjusted p < 0.01). Conversely, all individuals from the laboratory-susceptible reference strain (Lab) were wild-type homozygotes (8/8), with a mutant allele frequency of 0.

### 3.3 Correlation between *kdr* allele frequency and pyrethroid mortality

Quantitative assessment of the association between the *kdr* mutation and phenotypic resistance was performed by conducting a Pearson’s correlation analysis between the F1534S mutant allele (TCC) frequency and mortality rates for each pyrethroid across the 14 districts. A strong and statistically significant negative correlation was observed between the mutant allele frequency and mortality to permethrin (r = -0.85, df = 12, p < 0.001) and beta-cyfluthrin (r = -0.78, df = 12, p = 0.001). This indicates that districts with higher frequencies of the resistant *kdr* allele consistently exhibited lower mosquito mortality upon exposure to these insecticides. The correlation with alpha-cypermethrin was also negative but weaker and did not reach formal statistical significance (r = -0.52, df = 12, p = 0.056).

## 4 Discussion

This study provides a comprehensive assessment of pyrethroid resistance in *Ae. albopictus* populations across Hangzhou. Three pivotal findings emerge from this study: (i) field populations of *Ae. albopictus* throughout Hangzhou have developed widespread resistance to pyrethroid insecticides, particularly permethrin and beta-cyfluthrin; (ii) this resistance phenotype is strongly spatially correlated with a high frequency of the F1534S *kdr* mutation in the *VGSC* gene, which is nearly fixed in most populations; and (iii) both the prevalence of phenotypic resistance and *kdr* allele frequency exhibit significant geographical heterogeneity across different districts, and these two variables are spatially correlated at the population level.

### 4.1 High prevalence of pyrethroid resistance and the predominant role of F1534S *kdr*

The resistance profiles revealed by our bioassays are of substantial concern. Specifically, universal resistance to permethrin together with the high prevalence of resistance or possible resistance to beta-cyfluthrin seriously threatens the efficacy of pyrethroid-based vector control programs in Hangzhou. The molecular analyses provide a quantifiable explanation for the observed phenotype resistance. The exceptionally high frequency (89.23%) of the mutant TCC allele at the *VGSC* F1534 locus demonstrated a strong and statistically significant negative correlation with mortality rates at the district level for permethrin (r = -0.85, p < 0.001) and beta-cyfluthrin (r = -0.78, p = 0.001). While the robust population-level correlation, coupled with the known functional role of F1534S in reducing pyrethroid binding affinity, strongly supports the contribution of this mutation to resistance, a synergistic effect cannot be excluded since the metabolic detoxification enzymes were not examined. This finding aligns with the established role of F1534S as the most prevalent and dominant *kdr* mutation in *Ae. albopictus* across Asia, particularly Southeast Asia and regions in China such as Guangdong and Yunnan (Chen et al., 2021; Gao et al., 2018; Xu et al., 2016; Yang et al., 2021). The F1534S mutation, located in the IIS6 transmembrane segment of the *VGSC*, reduces the binding affinity of pyrethroids, thereby weakening the knockdown effect of the insecticide and reducing mortality (Dong et al., 2014). Notably, core urban districts (e.g., GS and YH) with the highest *kdr* allele frequencies (>98%) also exhibited the lowest mortality rates to permethrin (e.g., 1.33% in GS), providing robust local evidence for this genotype-phenotype relationship (Zhang et al., 2024; Zheng et al., 2022).

Conversely, all populations remained fully susceptible to bendiocarb (carbamate) and exhibited no confirmed resistance to fenitrothion (organophosphate). Furthermore, our extensive screening did not detect mutations at other major *kdr* loci (e.g., V1016G) or the G119 site of *ace-1*. This resistance profile indicates that the intensive use of pyrethroids in Hangzhou has exerted highly focused evolutionary selection pressure, leading to a relatively specific resistance mechanism. Although the potential contribution of metabolic resistance mechanisms (e.g., P450 monooxygenases) cannot be entirely excluded and may play a modulatory role, our results indicate that target-site insensitivity conferred by the F1534S mutation is the dominant mechanism underlying the observed pyrethroid resistance (Ishak et al., 2015).

### 4.2 Geographical heterogeneity: underlying drivers and implications

Taken together, these findings indicate that while the F1534S mutation has reached near-fixation in the central urban and peri-urban *Ae. albopictus* populations of Hangzhou, populations in more peripheral or less intensively managed districts retain higher proportions of susceptible alleles. The marked spatial heterogeneity in *kdr* allele distribution likely reflects differences in historical and current pyrethroid selection pressures across the city, thus highlighting the importance of localized resistance monitoring to guide vector control strategies.

A key contribution of this study is the identification of significant spatial heterogeneity in both resistance levels and the underlying *kdr* genetic structure. Specifically, populations from Fuyang District, Lin’an District, and Jiande City exhibited significantly lower *kdr* allele frequencies and correspondingly higher mortality rates compared to core urban districts such as Shangcheng District and Gongshu District. This geographical pattern likely reflects the intensity and history of pyrethroid application. Densely populated urban cores rely heavily on frequent, large-scale pyrethroid application for vector management and epidemic response, generating intense and continuous selection pressure that promotes the rapid fixation of resistance alleles (Karunamoorthi, 2011). Conversely, peri-urban and rural counties (e.g., Fuyang District and Lin’an District) may feature more diverse landscapes, including agricultural and semi-natural environments, where insecticide use may be less intensive or more diverse, thereby imposing weaker selection pressure for pyrethroid-specific *kdr* mutations (Zheng et al., 2022). Moreover, geographical and ecological barriers between these areas and the urban core may further restrict gene flow, facilitating the emergence of distinct local evolutionary trajectories (Kotsakiozi et al., 2017).

The remarkably high proportion of heterozygous individuals (50.00%) in Fuyang District is particularly informative. This population may represent a “frontline” in the spatial expansion of resistance, in which the frequency of the *kdr* mutation is actively increasing but has not yet reached fixation (Weetman et al., 2018). Such populations are critical early-warning indicators, highlighting areas where proactive intervention could delay or prevent the complete loss of pyrethroid efficacy.

Other environmental and ecological factors, apart from the extent of pesticide use, may contribute to the geographical heterogeneity of resistance. Urbanization gradients can lead to habitat fragmentation microclimate differences, and varying degrees of anthropogenic disturbances, thereby influencing mosquito population structure and the spread of resistance alleles (Newberry, 2025). Further, restricted migration and gene flow between mosquito populations may hinder the spatial dissemination of resistance alleles, especially in areas with significant geographical or ecological barriers (Barnes et al., 2017). Differences in land use types, such as, urban built-up areas, farmland, and natural forests may also indirectly regulate the rate of resistance development by affecting habitat suitability, pesticide exposure frequency, and population genetic structures (Ortiz et al., 2021; Vannier et al., 2022). Consequently, future spatial resistance models should integrate multi-source environmental data and population genomics information for comprehensive elucidation of the driving mechanisms underlying resistance heterogeneity.

### 4.3 Public health implications and resistance management recommendations

The pronounced geographical heterogeneity conveys a critical public health message: the resistance profile in a single location cannot be extrapolated to represent the resistance profile of the entirety of Hangzhou. Implementing city-wide control strategies based solely on data from highly resistant urban cores risks the unnecessary overuse of pyrethroids in more susceptible areas, potentially accelerating resistance spread. Conversely, strategies based on data from less resistant areas would be inadequate for effective control in the urban core, increasing the risk of mosquito-borne disease outbreaks. Accordingly, we advocate transitioning from a one-size-fits-all approach to a precision-based resistance management strategy (Organization, W. H and UNICEF, 2017).

We propose the following actionable recommendations: (i) Insecticide rotation and mixtures: in areas with confirmed high resistance, pyrethroids should be immediately and strictly rotated with alternative insecticide classes, specifically those that remained fully effective based on our findings, such as carbamates (e.g., bendiocarb) and other insecticides with low resistance risk. Furthermore, judicious use of insecticide mixtures with different modes of action should also be considered (Organization, W. H, 2023). (ii) Differentiated control measures: In “frontline” districts such as FY, enhanced surveillance should be prioritized. Vector control should emphasize non-chemical methods, such as environmental management and source reduction, to delay the fixation of *kdr* alleles and prolong pyrethroid susceptibility (van den Berg et al., 2021). (iii) Strengthened monitoring systems: Establishing a grid-based resistance-monitoring network encompassing the entire city is imperative. This system should integrate routine phenotypic bioassays with rapid molecular screening of *kdr* frequency to generate real-time, spatially specific data, forming an “insecticide resistance map” to guide precise, district-specific “one district, one policy” intervention strategies (Hancock et al., 2020).

### 4.4 Study limitations and future perspectives

A key methodological limitation of this study is that while the mosquitoes used for *kdr* genotyping were field-collected adults, those used in the insecticide bioassays were the F1 progeny of larvae from the same local populations. While this approach demonstrates a strong spatial correlation between the high frequency of the F1534S allele and widespread pyrethroid resistance at the population level, it does not establish a direct causal linkage between the genotype and the survival phenotype at the individual level. Future studies employing genotyping of mosquitoes that have survived diagnostic-dose or dose–response bioassays would be invaluable in confirming this causal relationship.

Secondly, the genotyping sample size in some regions was relatively small, which may have affected the accuracy of the estimated allele frequencies. While the main conclusions in this study are drawn based on overall data trends, interpretations for regions with small sample sizes should be made with caution. Future monitoring efforts should balance and increase sample sizes to improve the reliability of spatial heterogeneity analysis.

Thirdly, and most critically, this study did not assess metabolic resistance mechanisms. Cytochrome P450 monooxygenases, carboxylesterases, and glutathione S−transferases are well−documented contributors to pyrethroid resistance (Vontas et al., 2012) in Aedes mosquitoes and may substantially enhance the degree of resistance conferred by *kdr* mutations alone. The absence of synergist bioassays (e.g., with piperonyl butoxide, PBO) or enzyme activity assays leaves the relative contribution of metabolic versus target−site resistance unresolved in the Hangzhou populations. Future studies that prioritize an integrated mechanistic dissection, including, PBO synergism tests to infer P450−mediated resistance, biochemical quantification of detoxification enzyme activities (P450s, esterases, GSTs), and transcriptomic profiling (RNA−seq or RT−qPCR) to identify overexpressed metabolic genes and pathway signatures, are essential to address this gap.

Additionally, other lines of investigation will strengthen the resistance monitoring framework. These include, linking *kdr* genotypes to dose-response data that would enable quantitative assessment of the extent of resistance conferred by different genotypes (Brito et al., 2018). Further, resistance monitoring that incorporates such assays (e.g., establishing LC_50_/LC_90_ values) will aid accurate quantification of the actual levels of pyrethroid resistance in field populations. Finally, monitoring temporal changes in resistance allele frequencies under varying control strategies would help evaluate intervention efficacy and predict evolutionary trends (Hemingway, 2014). Functional validation of candidate resistance genes using approaches, such as, CRISPR/Cas9 gene editing could further clarify mechanistic networks underlying pyrethroid resistance.

## 5 Conclusion

In summary, this study provides evidence that, *Ae. albopictus* in Hangzhou has developed widespread resistance to pyrethroids. This phenomenon was observed at the population level and is spatially associated with a high-frequency F1534S *kdr* mutation, while also being characterized by significant geographical heterogeneity. The potential role of metabolic resistance mechanisms, which may exhibit geographical variation, was not investigated here and warrants further study. These findings suggest that a sole reliance on pyrethroids may become unsustainable. Consequently, these results underscore the potential need for an integrated resistance management strategy in Hangzhou. Such a strategy, informed by ongoing resistance monitoring and a deeper understanding of local resistance mechanisms (including the unevaluated metabolic factors), could involve insecticide rotation and area-specific interventions to mitigate resistance, preserve insecticide efficacy, and safeguard public health.

## Data Availability

The original contributions presented in the study are included in the article/**Supplementary Material**. Further inquiries can be directed to the corresponding authors.

## Supplementary material

Supplementary Table 1 Sampling points for Aedes albopictus in Hangzhou

Supplementary Table 2 Details of resistance levels of Aedes albopictus to five insecticides in different districts of Hangzhou

Supplementary Figure 1 Geographic distribution of *Ae. albopictus* sampling locations and F1534S allele frequencies in Hangzhou. Colored polygons represent administrative districts shaded according to the frequency of the F1534S (TCC) mutant allele (%). The color scale is divided into five intervals: 60–70%, 75–85%, 87–92%, 94–97%, and 97–100%. Black dots indicate individual sampling sites. Scale bar represents 10 and 20 miles.
